# Iatrogenic non-coronary leaflet perforation as a complication after robotic mitral valve repair

**DOI:** 10.1186/s13019-024-02753-4

**Published:** 2024-06-12

**Authors:** Afksendiyos Kalangos, Yilmaz Zorman, Emel Celiker Güler, Nataliia Shatelen

**Affiliations:** 1 Department of Cardiovascular Surgery, Koc University Hospital, Davutpasa Cd No:4, Istanbul, Topkapi, 34363 Turkey; 2 Department of Adult Cardiology, Koc University Hospital, Istanbul, Turkey; 3 Department of Cardiac Surgery, Heart Institute Ministry of Health of Ukraine, Kiev, Ukraine

**Keywords:** Mitral valve repair, Iatrogenic aortic regurgitation, Non-coronary leaflet perforation, Robotic mitral surgery

## Abstract

Iatrogenic aortic regurgitation secondary to leaflet injury is a rare complication of mitral valve surgery. For the first time, we report a patient who had progressive aortic regurgitation due to non-coronary leaflet perforation after robotic mitral valve repair and required aortic valve repair 18 months after this initial surgery. As in our case, aortic regurgitation after mitral valve surgery may remain undiagnosed on intraoperative transesophageal echocardiography or undetected until the patient’s discharge due to gradual enlargement of very small perforations over the postoperative course.

**Supplementary Information:**

The online version contains supplementary material available at 10.1186/s13019-024-02753-4.

The mitro-aortic intervalvular fibrosa ensures the continuity between the anterior mitral leaflet and mainly the left and non-coronary aortic leaflets and therefore plays an essential role in both valves’ anatomic and functional integrity [1]. Surgical sutures anchoring the mitral annuloplasty ring or prosthesis on the anterior annulus may cause left or non-coronary aortic leaflet injury [2–13] more often than that of the right leaflet [14], leading to aortic regurgitation (AR). As minimally invasive access to the mitral valve is becoming more widely used, this potential complication should be more carefully evaluated in the patients’ intraoperative, early, and midterm echocardiographic outcomes [9]. We report a case of iatrogenic AR in a patient who underwent robotic mitral valve repair (MVR). The patient’s informed consent was received.

A 39-year-old woman with a history of robot-assisted MVR for asymptomatic severe mitral regurgitation 18 months ago was first seen at our institution for surgical advice regarding the progressive AR detected on routine postoperative follow-up transthoracic echocardiographic (TTE) controls. The MVR consisted of an annuloplasty using a complete Medtronic CG Future ring (Medtronic, Inc, Minneapolis, USA), size 34. Interrupted 2 − 0 braided polyester nonpledgetted mattress sutures were placed robotically around the native mitral annulus for ring implantation.

The degree of AR on intraoperative transesophageal echocardiography (TEE) before MVR was estimated as trivial and as an eccentric mild to moderate leak after it, with no written information on its mechanism available in the patient’s medical file. She was discharged from the hospital on the fourth postoperative day with no residual mitral regurgitation or further aggravation of the aortic one. After discharge, the patient presented progressive dyspnea and palpitation episodes on moderate exertion, which gradually increased and became severe on the sixth postoperative month’s follow-up TTE. The last preoperative TEE showed severe eccentric AR, which seemed to originate from the non-coronary leaflet (Video 1). During the postoperative follow-up, the patient had no fever episodes or other clinical and biochemical factors in favor of suspected endocarditis.

The aortic valve was exposed through a transverse aortotomy and an 8-mm diameter hole with thickened fibrous circumference was found at the basal midpoint of the non-coronary leaflet (Fig. 1-A). The presence of braided mattress annuloplasty sutures incorporated into a fibrous reaction close to the annular extension of the hole was confirmed through the aortic orifice by exploring the mitro-aortic continuity. A bovine pericardial patch closed the perforation with a running 6/0 polypropylene suture material (Fig. 1-B). Intraoperative post-repair TEE confirmed satisfactory surgical correction with no residual leak. The postoperative course is uneventful over the two years after surgery.

**Fig. 1:**
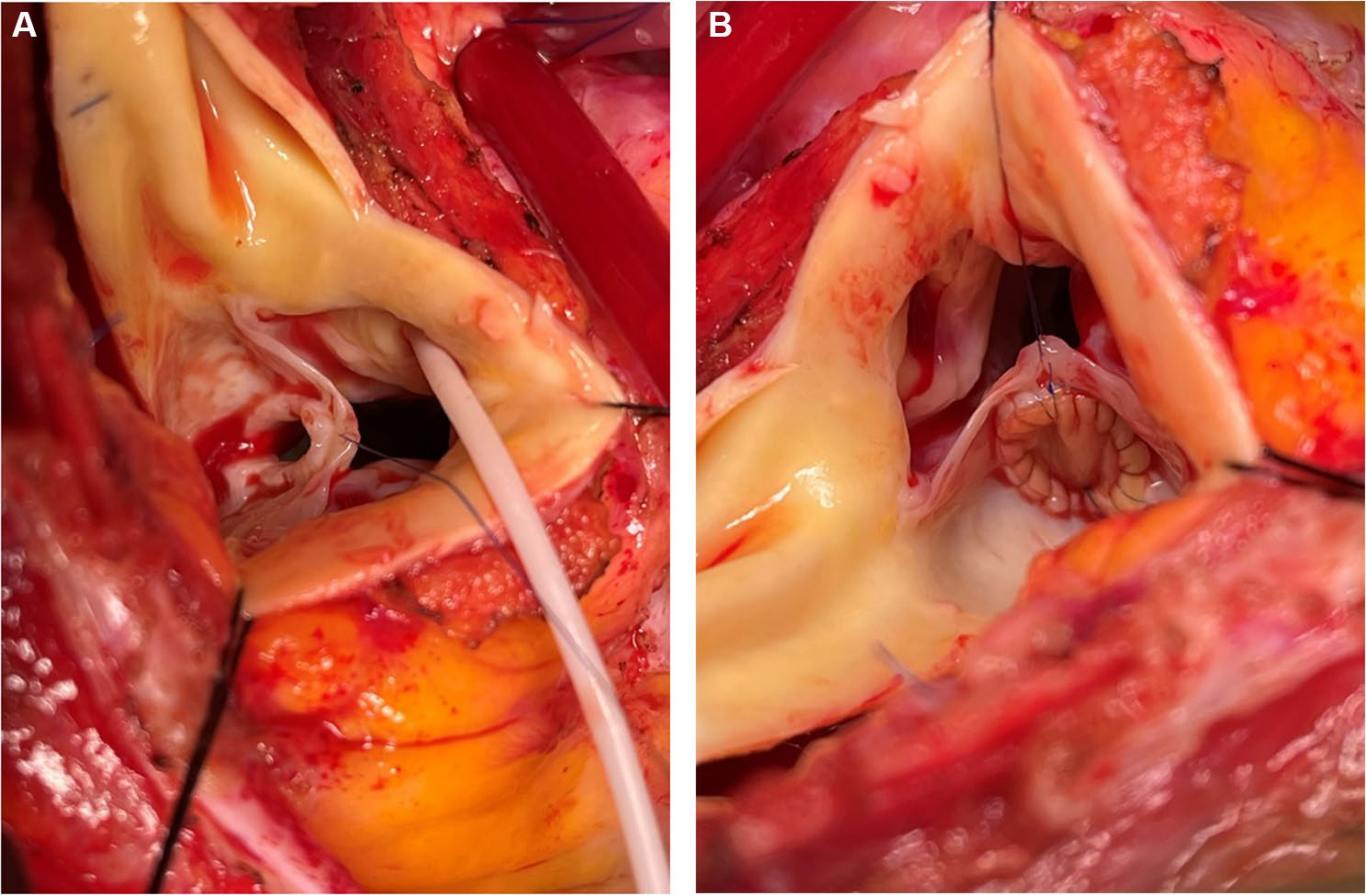
**A** Operative photograph of 8-mm non-coronary leaflet perforation at the midpoint of its basal portion. **B** Operative photograph of the perforation closed by a bovine pericardial patch

## Discussion

The anatomic continuity between the mitral and aortic valves is a fibrous, avascular, and fully dynamic portion of the heart that can potentially be the site of aortic valve injury during mitral annuloplasty or replacement [15]. Although surgeons practicing MVR in big-volume centers can be exposed to this complication during their professional career, the paucity of reported cases in the literature surprisingly makes us speculate that the proper number of iatrogenic aortic valve injury is underestimated. Aortic valve injury, especially of the left or non-coronary leaflet, usually occurs during the placement of the anterior mitral annuloplasty stitches while bringing the tip of 2 − 0 mattress braided sutures’ needle back from the left ventricular to the left atrial side across the anterior annulus. Partial rings can potentially decrease the risk of aortic valve injury as the portion of the anterior annulus between both trigonal areas does not necessitate any stitch placement. In all previously reported articles, AR resulted from tethering of left or non-coronary leaflet due to an inadvertently placed suture preventing proper cusp mobility [2, 4, 6, 7, 13] or perforation of one of the three aortic leaflets tackled by an improperly orientated needle during its passage through the anterior mitral annulus [3, 5, 8, 9, 11, 12, 14]. The non-coronary leaflet is more likely to suffer from injury than the left and right coronary leaflets. Out of the total 19 patients previously presented in the literature, 13 had injury of the non-coronary leaflet [2, 3, 8, 9, 11–13], 5 had that of the left coronary leaflet [3–7], and one had that of the right coronary leaflet [14]. In our case, the mechanism of progressive AR was probably due to the gradual increase of the non-coronary leaflet tear, as was previously described by Lakew et al. in three patients who underwent minimally invasive MVR [9]. Their patients gradually developed relevant AR over the postoperative course and required aortic valve repair 22 days, 6.5 months, and 4 years after their MVR [9]. Although advanced robotic technology enables better visualization of the annulus coupled with high definition and 3-dimensional secondary vision compared to minimally invasive techniques, the lack of tactile feedback in robotic surgery still persists, limiting the surgeon’s ability to assess suture depth, tension, and needle orientation [16].

In conclusion, the function of the aortic valve should be carefully checked on a routine basis on intraoperative post-repair TEE. The echocardiographer and the surgeon should seriously consider any change, even mild, in the degree of AR. In this condition, a better assessment of the mechanism of AR by intraoperative post-repair three-dimensional TEE should be adopted as a strategy [10]. If any potential aortic valve injury is suspected, exploration of the aortic valve at the time of the same surgery might be considered in conventional mitral valve procedures. The dilemma will persist in case of minimally invasive or robotic mitral procedures, whether or not post-repair AR changes have to impose the conversion of the incision.

### Electronic supplementary material

Below is the link to the electronic supplementary material.

Video 1. Preoperative two-dimensional TEE showing the aortic regurgitation through the non-coronary leaflet’s perforation.

## Data Availability

The data of the patient are available in the electronic medical file of the Koc University Hospital and can be accessed after the approval of the medical board of the Hospital.
